# Supplementary material for: In vivo generation of bone marrow from embryonic stem cells in interspecies chimeras
Source: eLife. 2022 Sep 30;11:e74018. doi: 10.7554/eLife.74018 (PMC9578712; doi:10.7554/eLife.74018)
Supplement: Supplementary file 1. [file elife-74018-supp1.docx]

**Supplementary file 1.** The number and percentage of hematopoietic BM cells containing both mouse and rat mRNAs (hybrid cells).

| **Cluster** | **Cell number** | **Cells with mouse and rat mRNAs** | **Frequency (%)** |
| --- | --- | --- | --- |
| Lymphoid progenitor | 199 | 0 | 0 |
| Pro.B cell | 156 | 0 | 0 |
| Pre.B cell | 541 | 0 | 0 |
| B cell | 808 | 4 | 0.0049505 |
| T cell | 243 | 0 | 0 |
| Erythroid progenitor | 1122 | 1 | 0.00089127 |
| Megakaryocyte | 101 | 0 | 0 |
| Myeloid progenitor | 465 | 0 | 0 |
| Basophil-Eosinophil | 543 | 0 | 0 |
| Dendritic | 419 | 0 | 0 |
| Monocyte | 165 | 0 | 0 |
| Neutrophil | 300 | 1 | 0.00333333 |
| Neutrophil progenitor | 358 | 0 | 0 |
| Lineage-negative | 598 | 0 | 0 |
