# Supplementary material for: In vivo generation of bone marrow from embryonic stem cells in interspecies chimeras
Source: eLife. 2022 Sep 30;11:e74018. doi: 10.7554/eLife.74018 (PMC9578712; doi:10.7554/eLife.74018)
Supplement: Supplementary file 2. [file elife-74018-supp2.docx]

**Supplementary file 2.** The number of counts and features (genes) in 6 hybrid cells identified in mouse-rat chimera.

| **Cell barcode** | **Identity of cells based on mouse transcriptome** | **Mouse_**  **nCount** | **Mouse_**  **nFeature** | **Rat_**  **nCount** | **Rat_**  **nFeature** |
| --- | --- | --- | --- | --- | --- |
| AAGACTCTCGACATTG-1 | B cell | 5286 | 2009 | 2044 | 889 |
| AGAAGTAAGCAGGGAG-1 | B cell | 4575 | 1736 | 14761 | 3340 |
| AGGGTCCGTCTTGGTA-1 | Neutrophil | 8428 | 1622 | 931 | 552 |
| CCGGTGACAGTGGCTC-1 | Erythroid progenitor | 43228 | 5455 | 3955 | 1415 |
| CTAACTTTCCAATCCC-1 | B cell | 3735 | 1721 | 1155 | 689 |
| CTCAACCTCAACTCTT-1 | B cell | 6018 | 2225 | 2320 | 1192 |
