# Supplementary material for: In vivo generation of bone marrow from embryonic stem cells in interspecies chimeras
Source: eLife. 2022 Sep 30;11:e74018. doi: 10.7554/eLife.74018 (PMC9578712; doi:10.7554/eLife.74018)
Supplement: Supplementary file 3. [file elife-74018-supp3.docx]

**Supplementary file 3.** Antibodies used for Flow Cytometry (FC) and Immunofluorescence staining (IF).

| **Antibody** | **Method** | **Manufacturer** | **Catalog No.** | **Dilution** |
| --- | --- | --- | --- | --- |
| Lineage- | FC | BD Bioscience | 561317 | 1:100 |
| CD117 | FC | Thermo Fisher | 12-1171-83 | 1:100 |
| Sca1 | FC | Thermofisher | 17-5981-81 | 1:100 |
| CD150 | FC | Thermo Fisher | 25-1502-80 | 1:100 |
| CD45R | FC | Thermo Fisher | 83-0452-41 | 1:100 |
| Gr1 | FC | Biolegend | 108433 | 1:100 |
| CD3e | FC | Biolegend | 100312 | 1:100 |
| CD11b | FC | Biolegend | 101216 | 1:100 |
| Ter119 | FC | Biolegend | 116228 | 1:100 |
| CD48 | FC | Thermo Fisher | 48-0481-82 | 1:100 |
| FLK1 | IF | Santa Cruz Biotech. | Sc-393163 | 1:200 |
| RUNX1 | IF | Abcam | Ab92336 | 1:500 |
