# Supplementary material for: In vivo generation of bone marrow from embryonic stem cells in interspecies chimeras
Source: eLife. 2022 Sep 30;11:e74018. doi: 10.7554/eLife.74018 (PMC9578712; doi:10.7554/eLife.74018)
Supplement: Transparent reporting form [file elife-74018-transrepform1.pdf]

Westbrook Centre
Milton Road
Cambridge CB4 1YG, UK

P 01223 855340
W elifesciences.org
T @elife

eLife's transparent reporting form

We encourage authors to provide detailed information *within their submission* to facilitate the interpretation and replication of experiments. Authors can upload supporting documentation to indicate the use of appropriate reporting guidelines for health-related research (see EQUATOR Network), life science research (see the BioSharing Information Resource), or the ARRIVE guidelines for reporting work involving animal research. Where applicable, authors should refer to any relevant reporting standards documents in this form.

If you have any questions, please consult our Journal Policies and/or contact us: editorial@elifesciences.org.

Sample-size estimation

- You should state whether an appropriate sample size was computed when the study was being designed
- You should state the statistical method of sample size computation and any required assumptions
- If no explicit power analysis was used, you should describe how you decided what sample (replicate) size (number) to use

Please outline where this information can be found within the submission (e.g., sections or figure legends), or explain why this information doesn't apply to your submission:

Statistical significance was determined using one-way ANOVA and Student's t-test. P ≤ 0.05 was considered statistically significant. N numbers and p values are provided in every figure legend.
1) Weights of mouse-rat chimeras are shown at different time points and compared to rats and mice of similar ages. n=7-18 were used in each group. No outliers have been excluded in the analysis.
2) Flow cytometry experiments were performed using 8-16 animals per group.
3) Survival studies were performed using n=12-20 animals in each group.
4) Single-cell RNA sequencing of ESC-derived BMCs in P10 pups were performed using 3 animals in each group.
5) Single-cell RNA sequencing of ESC-derived BMCs and bone cells in P5 pups were performed using 5 animals in each group.

Replicates

- You should report how often each experiment was performed
- You should include a definition of biological versus technical replication
- The data obtained should be provided and sufficient information should be provided to indicate the number of independent biological and/or technical replicates
- If you encountered any outliers, you should describe how these were handled
- Criteria for exclusion/inclusion of data should be clearly stated
- High-throughput sequence data should be uploaded before submission, with a private link for reviewers provided (these are available from both GEO and ArrayExpress)

Please outline where this information can be found within the submission (e.g., sections or figure legends), or explain why this information doesn't apply to your submission:

eLife Sciences Publications, Ltd is a limited liability non-profit non-stock corporation incorporated in the State of Delaware, USA, with company number 5030732, and is registered in the UK with company number FC030576 and branch number BR015634 at the address Westbrook Centre, Milton Road, Cambridge CB4 1YG.

The number of all biological replicates were provided in each Figure legend. No outliers have been excluded in the analysis.

Statistical reporting

- Statistical analysis methods should be described and justified
- Raw data should be presented in figures whenever informative to do so (typically when N per group is less than 10)
- For each experiment, you should identify the statistical tests used, exact values of N, definitions of center, methods of multiple test correction, and dispersion and precision measures (e.g., mean, median, SD, SEM, confidence intervals; and, for the major substantive results, a measure of effect size (e.g., Pearson's r, Cohen's d)
- Report exact p-values wherever possible alongside the summary statistics and 95% confidence intervals. These should be reported for all key questions and not only when the p-value is less than 0.05.

Please outline where this information can be found within the submission (e.g., sections or figure legends), or explain why this information doesn't apply to your submission:

This information was provided in figure legends and at the end of Materials and Methods. Statistical significance was determined using one-way ANOVA and Student's t-test. Multiple means were compared using one-way analysis of variance with the post-hoc Tukey test. $P \leq 0.05$ was considered statistically significant. For datasets with n<5, non-parametric Mann-Whitney U test was used to determine statistical significance. Data were presented as mean ± standard error of mean (SEM).

(For large datasets, or papers with a very large number of statistical tests, you may upload a single table file with tests, Ns, etc., with reference to sections in the manuscript.)

Group allocation

- Indicate how samples were allocated into experimental groups (in the case of clinical studies, please specify allocation to treatment method); if randomization was used, please also state if restricted randomization was applied
- Indicate if masking was used during group allocation, data collection and/or data analysis

Please outline where this information can be found within the submission (e.g., sections or figure legends), or explain why this information doesn't apply to your submission:

Animals were allocated into experimental groups based on genotype, and information was provided in figure legends and at the end of Materials and Methods.

Additional data files ("source data")

- We encourage you to upload relevant additional data files, such as numerical data that are represented as a graph in a figure, or as a summary table
- Where provided, these should be in the most useful format, and they can be uploaded as "Source data" files linked to a main figure or table
- Include model definition files including the full list of parameters used
- Include code used for data analysis (e.g., R, MatLab)
- Avoid stating that data files are "available upon request"

eLife Sciences Publications, Ltd is a limited liability non-profit non-stock corporation incorporated in the State of Delaware, USA, with company number 5030732, and is registered in the UK with company number FC030576 and branch number BR015634 at the address Westbrook Centre, Milton Road, Cambridge CB4 1YG.

Please indicate the figures or tables for which source data files have been provided:

All bone marrow scRNA-seq data were uploaded to the Gene Expression Omnibus (GEO) database under accession number GSE184940.
